# The implications of oncolytic viruses targeting fibroblasts in enhancing the antitumoural immune response

**DOI:** 10.1016/j.heliyon.2024.e39204

**Published:** 2024-10-10

**Authors:** Ibrahem Al-Obaidi, Ciaran Sandhu, Bilal Qureshi, Leonard W. Seymour

**Affiliations:** a Department of Oncology, University of Oxford, Old Road Campus Research Building, Roosevelt Drive, Oxford, OX3 7DQ, UK; b The Queen's College, High Street. Oxford, OX1 4AW, UK; c Somerville College, Woodstock Road, Oxford, OX2 6HD, UK

**Keywords:** Cancer-associated fibroblast, Oncolytic virus, Anti-tumoural immunity, Immunotherapy

## Abstract

Oncolytic viruses (OVs) are an emerging immunotherapy platform that selectively target tumour cells, inducing immunogenic cell death. This reverses the ‘immune-desert’ phenotype of tumours, enhancing antitumour immunity. However, oncolytic virotherapy has shown limited efficacy in solid tumours due to the presence of protumoural, immunosuppressive cancer-associated fibroblasts (CAFs). Recent studies have explored OVs that specifically target CAFs to enhance antitumoural immune responses, with promising results. Nevertheless, detailed interrogation of the experimental design of these studies casts doubt on their potential for successful clinical translation. Most studies targeted CAFs non-specifically, failing to acknowledge CAF heterogeneity, with antitumoural CAFs also present. Thus, use of transcriptomics is advisable to provide more focused targeting, limiting potential off-target toxicity. Furthermore, experiments to date have largely been conducted in murine models that do not faithfully recapitulate tumour microenvironments, potentially biasing the efficacy observed. Future work should make use of humanised patient-derived xenograft murine models for animal studies, after which primary human tumour biopsies should be utilised to more closely represent the patient population for maximal translation relevance. Additionally, approaches to enhance the antitumoural immune responses of this therapy should be prioritised, with the ultimate aim of achieving complete remission, which has not yet been observed pre-clinically.

## 1 Introduction

Oncolytic viruses (OVs) are an emerging immunotherapy platform used to treat patients with neoplastic lesions, with four OVs currently approved [1]. These viruses selectively target tumour cells [2], either due to natural tropism or genetic modification to exploit the tumour phenotype, such as expressing viral genes under tumour-specific promoters [3]. This reduces off-target toxicity and hence provides OVs with a tolerable safety profile superior to other immunotherapy approaches. Selective replication in and lysis of tumour cells results in immunogenic cell death due to the release of viral pattern-associated molecular patterns (PAMPs) and tumour cell damage-associated molecular patterns (DAMPs), as well as neoantigens from damaged tumour cells, which in turn stimulate dendritic cell maturation. These subsequently produce pro-inflammatory cytokines such as IL-2 and TNF-α and hence promote tumour-associated antigen-specific T-cell activation [4], as well as chemokines such as CCL2 and CXCL10. This results in reversal of the ‘immune-desert’ phenotype of immunoedited tumours through attraction of other immune cells such as CD8^+^ T-cells, B-cells and tumour-associated macrophages, enhancing antitumour immunity. However, OVs showed limited response as a monotherapy [5], necessitating amplification of efficacy through utilisation of OVs programmed to express multiple transgene “payloads” upon replication in tumour cells, including bispecific T-cell engagers (BiTEs) [6], immune checkpoint blockade antibodies [7] and immunomodulatory cytokines [8]. Such combinatorial therapies have shown promising synergistic effects in both preclinical studies and clinical trials [[9], [10], [11]].

**Fig. 1:**
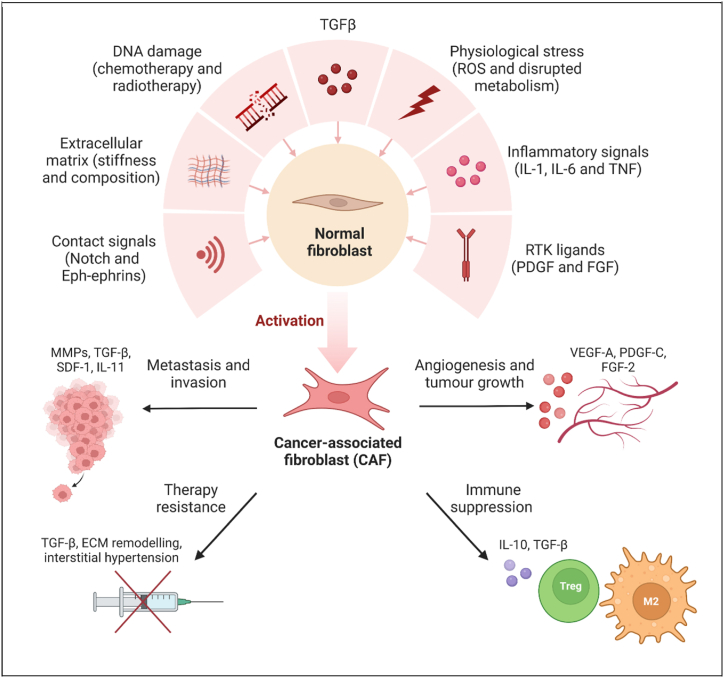
– Schematic of CAF activation and their protumoural effects. Adapted from “Mechanisms of Cancer-associated Fibroblast Activation”, by BioRender.com (2024). Retrieved from https://app.biorender.com/biorender-templates.

Importantly, current OV strategies largely neglect the tumour stroma, which can comprise 90 % of the tumour mass [12]. This stroma is thought to have thus far limited the efficacy of oncolytic virotherapy against solid tumours [13] due to the stromal physical barrier preventing effective penetration of OVs and immune cells, hence contributing to the immunosuppressive TME. One of the major constituents of the tumour stroma are the cancer-associated fibroblasts (CAFs) [14,15]. These are a heterogenous group of activated fibroblasts, consisting of both pro- and anti-tumoural subtypes. Although we lack a precise understanding of their origin [16], CAFs are thought to be normal fibroblasts activated in the TME through different mechanisms as part of the wound-healing response to the cancer “wound” (Fig. 1) [17]. These activated CAFs mediate many pro-tumourigenic functions that have limited the efficacy of OVs (Fig. 1).This includes secreting extracellular matrix (ECM), which results in an increased interstitial pressure within tumours, from negligible in normal tissues to over 15 mmHg in many tumours [18]. This hinders the spread of OVs and hence reduces their ability to infect other tumour cells [3]. CAFs also secrete more cytokines and chemokines than normal fibroblasts, including anti-inflammatory TGF-β and VEGF, as well as recruiting other immunosuppressive cells such as Tregs and M2 macrophages. This overall promotes immunosuppression [19], angiogenesis and metastasis, countering OV effects. Despite this, subtypes of CAFs have also been shown to mediate anti-tumourigenic functions, albeit less well described. This includes suppressing regulatory T-cells and maintaining CD8^+^ functionality [20], and hence having the potential to enhance the efficacy of OVs. As these CAFs are relatively genetically stable compared to the more heterogenous tumour cells [21], this makes them an appealing target for improving the antitumour immune response induced by OVs. Crucially, the heterogeneous nature of CAFs warrants specific targeting of protumoural CAFs in order to shift the equilibrium in favour of antitumoural CAFs thereby potentiating tumour suppression and improve treatment outcomes.

It is hypothesised that utilising CAF-targeting OVs can result in an enhanced antitumoural immune response. These OVs are often bioselected or genetically engineered to target markers expressed on CAFs, in contrast to traditional OVs that exploit molecular signatures unique to the tumour cells. This new approach has been explored in many preclinical studies, with increased antitumoural responses being emphasised [21,22]. However, previous methods of targeting CAFs have been largely unsuccessful in clinical trials [23,24]. This includes an anti-FAP monoclonal antibody Sibrotuzumab [23], which showed promise in preclinical studies. Given the complex heterogeneity of the fibroblast population in tumours, as well as the limitations of preclinical models, it is likely that the CAF-targeting OVs used in these studies will similarly encounter difficulty in translation to the clinic. As such, this review will aim to.

1) Summarise the current evidence regarding OVs targeting fibroblasts in enhancing antitumoural responses.
2) Unravel the limitations of these studies that may hinder their translation to the clinic.
3) Propose suggestions for future studies within the field.

### 1.1 Oncolytic viruses targeting fibroblasts: the current evidence

To date, there have been several studies that have utilised OVs to target CAFs (Table 1). Mechanisms of targeting include exploiting natural CAF tropism, bioselection through repeated CAF-passage of a rapidly mutating adenovirus, or genetic modification to express transgene payloads such as CAF-targeting BiTEs. Studies have utilised different CAF markers for targeting, notably fibroblast activation protein (FAP) and junctional adhesion molecule-A (JAM-A). Whilst these studies have limitations (Table 1), an enhancement of the overall antitumour immune response has been observed, inducing a pro-inflammatory TME and enhancing T-cell mediated killing of tumour cells, emphasising reversal of CAF-mediated immunosuppression [25]. CAF targeting has also been shown to disrupt the stromal barrier, emphasised by Puig-Saus et al. [26,27] with increased plaque size *in vitro* of the bioselected adenovirus, highlighting increased intratumoural spread of OVs. BiTE-mediated redirection of T-cells towards CAFs has also been thought to delay viral clearance through reducing anti-viral immunity, a substantial issue currently limiting OV spread and efficacy. Such promise has facilitated the clinical assessment of enadenotucirev adenovirus encoding anti-FAP/CD3-BiTE [25].

**Table 1:** Summary of the preclinical studies examining CAF targeting by OVs.

Study	OV type	Mechanism of targeting	Key outcomes	Limitations
Jing et al., 2017 [28]	Genetically-engineered measles virus	Virus replicates in murine or human uPAR positive tumour and stromal cells, in a species-specific manner.	Combination of both murine and human uPAR-targeting OVs outperformed monotherapy in a murine orthotopic xenograft breast cancer model with murine stromal cells and human tumour cells.	uPAR is not uniquely expressed on tumour and stromal cells, hence specific effects of CAF loss cannot be determined.
Yu et al., 2017 [29]	Genetically-engineered vaccinia virus	Virus replicates within tumour cells and expresses an anti-FAP BiTE (mFAP-TEA-VV) on tumour cell replication.	In a B16 melanoma mouse model, mFAP-TEA-VV showed greater reduction in tumour growth compared to control OVs targeting only tumour cells.	FAP is expressed by other stromal cells. Tumour cells were grown over a timespan of a few days, hence they may not recapitulate the level of tumour heterogeneity characteristic of typical human tumours.
Puig-Saus et al., 2012 [26], 2014 [27]	Bioselected adenovirus (Ad)	Virus replicates in CAFs and tumour cells, with release potentiated by the iLG397T i-leader truncation mutation.	In an immunocompetent syngeneic Syrian hamster pancreatic tumour model, OV spread measured by plaque size *in vitro* was increased by iLG397T Ad targeting of both tumour cells and CAFs.	A specific pancreatic carcinoma cell line was used with enhanced permissiveness to Ad replication. Both CAFs and tumour cells were targeted, hence specific effects of CAF loss cannot be determined.
Lopez et al., 2009 [30]	Genetically-engineered adenovirus	Virus selectively replicates inSPARC-expressing cells, including CAFs and tumours.	Infection with Ad-F512 in xenograft immunocompromised mice with human SB2 melanoma cells and WI-38 foetal fibroblasts delayed tumour growth to a lesser extent than xenografts of melanoma cells alone.	Both fibroblasts and tumour cells were targeted, hence specific effects of CAF loss cannot be determined Foetal fibroblasts differ phenotypically from CAFs.
Freedma-n et al., 2018 [25]	Genetically-engineered enadenotucirev adenovirus (EnAd)	Virus replicates within tumour cells and expresses an anti-FAP BiTE (EnAd-FAP-BiTE) on tumour cell replication.	EnAd-FAP-BiTE activated T-cells in malignant ascites and solid prostate cancer tissue clinical biopsies, depleting FAP^+^ cells and reducing TGF-β. Measuring changes in gene expression highlighted upregulation of pro-inflammatory cytokines, T-cell function and T-cell attractant chemokines.	FAP is expressed by other stromal cells, so selective targeting of CAFs is not certain. Potential loss of tissue architecture in biopsies during storage.
De Sostoa et al., 2019 [31]	Genetically-engineered adenovirus	Virus replicates within tumour cells and expresses an anti-FAP BiTE (ICO15K-BiTE) on tumour cell replication.	Enhanced T-cell activation and accumulation was observed in human lung or pancreatic cell line-derived xenograft NSG murine models treated with ICO15K-BiTE compared with controls. Reduced tumour growth was observed, associated with depletion of FAP-expressing cells. No significant off-target toxicity was detected.	Immunocompromised mice were used. FAP is expressed by PBMCs other than T-cells, so effect may not be due to targeting CAFs. *In vitro,* cytokines levels induced were higher with murine FAP^+^ cells compared to human FAP^+^ cells, hence efficacy in this study may be unrepresentative of humans.
Harryvan et al., 2022 [32]	Bioselected/naturally tropic reovirus	Each viral strain selectively replicates in junctional adhesion molecule-A (JAM-A) expressing fibroblasts.	Infection and apoptosis was observed in human and mouse JAM-A positive pancreatic ductal adenocarcinoma cancer (PDAC)-derived fibroblasts. Reoviral infection decreased cell viability in organoids of human and murine PDAC cells with JAM-A positive and negative fibroblasts.	No assessment of antitumoural immunity. The proportion of JAM-A positive to negative fibroblasts may differ from organoids to tumours.
Kurisu et al., 2021 [33]	Naturally tropic reovirus	Virus selectively replicates in JAM-A CAFs.	Reovirus infection in a B16 murine tumour model was associated with upregulation of pro-inflammatory genes (including interferon-stimulated genes) in CAFs, αSMA^+^ CAF apoptosis and tumour cell death.	Greater degree of tumour cell apoptosis than loss of CAFs, hence the cannot be solely attributed to CAF loss. No human CAFs used. No assessment of antitumoural immunity
Mistarz et al., 2023 [34]	Genetically-engineered vaccinia virus	Virus replicates in CAFs and expresses a CXCR4 antagonist (OXCXCR4-A).	CXCR4 antagonism led to depletion of CAFs, M1 polarisation and CD8^+^ T-cell responses to SV40 T-antigen positive tumours in an orthotopic murine ovarian carcinoma model.	No assessment of human CAFs.

This picture of OVs depleting CAFs is however complicated by contradictory evidence suggesting that CAFs may be essential for enhancing OV efficacy in inducing antitumour immune responses. Cross-talk between CAFs and tumour cells was shown to sensitise them to vesicular-stomatitis virus infection [35]. This was shown to be mediated by TGF-β released from tumour cells and FGF2 released from CAFs, both of which downregulated antiviral immune responses, including interferon responses and RIG-I, and hence increased OV intratumoural spread [35]. Other studies have further highlighted a potential role for soluble factors secreted by CAFs in enhancing OV functionality, with conditioned media from fibroblasts enhancing OV activity in pancreatic MIA PaCa-2 cancer cells [30].

Evidence for immunosuppressive and antitumoural effects of CAFs, which limit and enhance OV efficacy respectively, casts doubt on the success of these therapies in the clinic. This is due to potential depletion of antitumoural CAFs that may worsen outcome and patient's responses, a problem encountered previously by anti-CAF therapies [36]. Thus, successful translation will likely require rational study design to account for these potential pitfalls moving forward.

### 1.2 Challenges for translation to the clinic

#### 1.2.1 Heterogeneity of the CAF population

CAFs represent a highly heterogenous population of cells, with differing phenotypes depending on their location and local signalling factors within the TME. These CAFs are defined by a lack of expression of epithelial, endothelial or haematopoietic biomarkers, and a combination of many different tentative mesenchymal biomarkers, including FAP, αSMA and PDGFRα [17]. A variety of techniques have been utilised to elucidate distinct CAF subpopulations, including technologies such as single-cell RNA sequencing (scRNA-seq) and mass cytometry by time-of-flight (CyTOF) [37] (Table 2). Notably, such analysis has been largely conducted in pancreatic cancers, and hence may not be representative of all cancer CAF populations.

**Table 2:** – Summary of the different CAF subtypes. ↑, upregulation; ↓, downregulation; →, leading to; CAF, cancer-associated fibroblasts; myCAFs, myofibroblastic CAFs; iCAFs, inflammatory CAFs; cCAFs, circulating CAFs; apCAFs, antigen-presenting CAFs; CD10^+^GRP77^+^ CAFs, CD10^+^ G-protein-coupled receptor 77 CAFs; FAP, fibroblast-associated protein; αSMA, α-smooth muscle actin; VIM, vimentin; COL1A1, collagen type I alpha I chain; PDPN, podoplanin; DCN, decorin; TAGLN, transgelin; TPM1/2, tropomyosins 1 and 2; AGTR1, angiotensin II receptor type 1; DPT, dermatopontin; LMNA, laminin A/C; MHC-II, major histocompatibility complex class II; H2-Ab1, histocompatibility 2 class II antigen A and beta1; PDGFRβ, platelet-derived growth factor receptor beta; MYL9, myosin light chain 9; IL-6, interleukin-6; IL-8, interleukin-8; G-CSF, granulocyte-colony stimulating factor; ECM, extracellular matrix; SLPI, secretory leukocyte peptidase inhibitor; NF-κB, nuclear factor kappa B.

CAF subtype	Effect on tumour	Biomarkers	Characteristics
myCAFs [38,39]	Uncertain	FAP, αSMA, VIM, COL1A1, PDPN, DCN, TAGLN, TPM1/2	Contractile protein expression (TAGLN, TPM1/2, MYL9) ↑Collagen production
iCAFs [38]	Likely protumoural	FAP, VIM, COL1A1, PDPN, DCN, AGTR1, DPT, LMNA	Secrete inflammatory mediators: IL-6, IL-8, G-CSF ↑ECM synthesis → impairs vasculature → therapeutic resistance
cCAFs [40]	Protumoural	FAP, COL1A1, VIM	Circulate with tumour cell clusters Involved in metastasis and forming pre-metastatic niches
apCAFs [41]	Uncertain	FAP, VIM, COL1A1, DCN, PDPN, ↓co-stimulatory molecules (CD80/86), MHC-II (H2-Ab1)	MHCII expression without co-stimulatory molecules → anergy of CD4^+^ T-cells SLPI → enhance proliferation of myeloid cells
CD10^+^GRP77^+^ CAFs [42]	Protumoural	FAP, αSMA, PDGFRβ, CD10, GPR77	↑NF-κB, IL-6 and IL-8 → chemoresistance and cancer stemness

The surface markers currently utilised to target CAFs are both not CAF-specific and do not differentiate between CAF subtypes. For example, FAP is expressed on macrophages [43], as well as on both anti- and protumoural CAFs (Table 2). As such, whilst these treatments have shown varying degrees of success in preclinical models, off-target effects are likely to be observed in future trials, such as the depletion of antitumoural CAFs, mediating tumour growth. This may account for the discrepancy in outcome of targeting CAFs in preclinical models of PDAC. Although myCAF populations have been implicated as antitumoural in studies of PDAC organoids [38], reduction in animal survival in a murine model for pancreatic cancer after depleting the “immunosuppressive” αSMA^+^ myCAFs was observed [39]. This heterogeneity highlights the need for description of the specific functional CAF phenotype involved in the studies in Table 1, which is currently lacking.

**Fig. 2:**
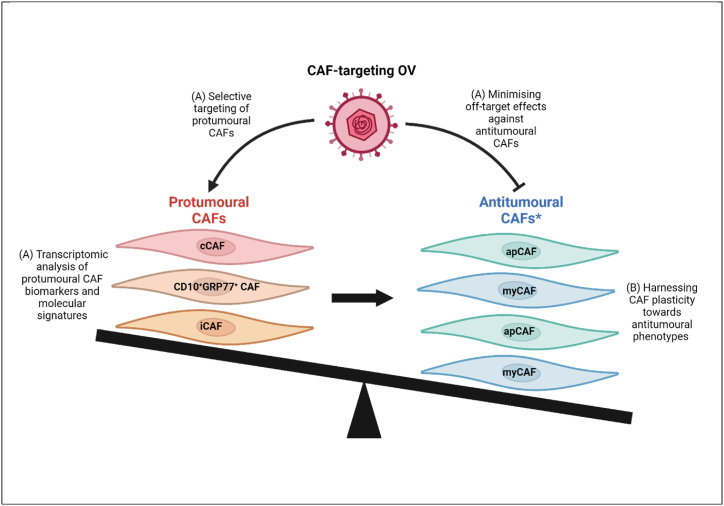
Potential avenues towards optimising CAF-targeting OV design to shift the equilibrium of the TME in favour of antitumoural CAFs. (A) Recognition of CAF heterogeneity through selective targeting of protumoural CAFs and minimising off-target effects towards antitumoural CAFs. Exposure of the extent of CAF-heterogeneity may be uncovered through multi-omic approaches, especially transcriptomic analysis for biomarkers and molecular signatures within the recognised CAF subtypes. (B) Exploiting factors that facilitate plasticity of protumoural CAF subtypes towards antitumoural subtypes. ∗Antitumoural CAFs are those in which antitumoural activity has been observed although their predominant phenotype is undetermined. Created in BioRender.com.

Future CAF-targeting OV studies should undertake transcriptomic analysis, as recently conducted by Mistarz et al. [34], for identification of the immunomodulatory phenotype of different subpopulations of CAFs in these studies. However, making use of spatially-resolved transcriptomics is recommended as this makes use of histological tissue, reducing the detachment of fibroblasts from tissues that could alter their phenotype, such as myCAFs requiring contact to maintain their phenotype in PDAC organoids [38]. Overall, this would increase our understanding of the expression of pro- and anti-inflammatory factors to delineate CAF function, as well as categorise CAFs in specific tumours for depletion by OVs based on their specific biomarkers and molecular pathways, such as LRRC15^+^ protumoural myCAFs identified using scRNA-seq of murine and human PDAC samples [36]. This would aid in overcoming the current limitation of depletion based on general CAF biomarkers until a pan-specific marker for certain subtypes is established, as current studies [39] have emphasised depletion of CAFs has resulted in tumour progression rather than suppression, raising caution regarding non-specific CAF depletion for treatment outcomes. Transcriptomic assessment of CAFs from tumour biopsies would enable CAF subtype identification, such as through the biomarkers identified in Table 1, as well as reveal differing signalling pathways and molecular signatures associated with a protumourigenic phenotype. This would allow for preferential OV targeting of protumoural CAFs (Fig. 2) to direct TMEs towards tumour suppression, potentially improving treatment outcomes, where previous non-specific CAF targeting strategies such as Sibrotuzumab have failed. This uniform analysis of CAFs would also allow greater extrapolation of findings for future CAF-targeting OV studies, driving progress within the field.

#### 1.2.2 CAF plasticity

CAFs have been shown to exhibit phenotypic plasticity on alteration of environments, with iCAFs cultured in PDAC organoids able to revert to a myCAF phenotype lacking inflammatory cytokine expression when plated as a monolayer. This may present an issue for clinical application. Both T-cells targeting CAFs through BiTE-expressing OVs and general increase in immune-infiltrate could lead to modulation of the phenotype of CAFs through immunoselection of more protumoural, immunosuppressive CAFs. This could be through cross-talk with tumour cells or interactions with immune cells. For example, co-culturing NK cells with CAFs has been shown to result in increased expression of PGE2 by CAFs, which itself inhibits NK cell activity, forming an immunosuppressive loop [44,45]. Such alteration in CAF phenotype may not have been detected in current studies, both due to a lack of CAF functionality measurement and because mice tend to have far fewer CAFs than humans and hence not enough time for immunoselection**.** Future studies should investigate this potentially harmful effect as there is a paucity of literature surrounding this, as well as ways of exploiting CAF plasticity to evoke antitumoural CAF phenotypes (Fig. 2). This could be done by comparing gene and protein expression profiles of CAFs at different time points post-administration of CAF-targeting OVs to highlight any phenotypic alterations, as well as potentially identifying targetable pathways which would favour transition to an antitumoural CAF phenotype. Furthermore, the individual impact of immune cell populations on CAFs can be assessed by depleting specific immune cells, genetically or via monoclonal antibodies, in immunocompetent mice prior to OV administration.

#### 1.2.3 Murine models

The majority of preclinical studies investigating OV-targeting CAFs have made use of murine models (Table 3). However, this has several limitations that could impair evaluation of oncolytic virotherapy. Immunocompromised mouse strains, whilst enabling engraftment of human tumour cells, do not exhibit comparable levels of antiviral immunity which normally limits OV spread in humans [1]. This could bias results to appear more antitumoural. This has been overcome in some studies via implementation of immunocompetent hosts, allowing for more accurate representation of tumour immune infiltration and immune responses. However, immune responses tend to be species-specific [46] due to the significant differences between mice and human immune systems, including cytokines, lymphocyte signalling pathways and chemokine receptor expression and a greater preponderance of lymphocytes compared to humans [47]. As such, future studies would benefit from using humanised bone marrow-liver-thymus (BLT) mouse models [48] to reconstitute the human immune system within mice for more representative immune responses.

**Table 3:** – Description of the animal models used in the experimental studies utilising CAF-targeting OVs.

Study	Sex of animal model	Tumour type	Type of model used
Jing et al., 2017 [28]	Female mice	Breast	Immunocompromised orthotopic human cell line-derived xenograft
Yu et al., 2017 [29]	Unspecified	Melanoma	Immunocompetent subcutaneous syngeneic model
Puig-Saus et al., 2014 [27]	Female Syrian hamster	Lung and pancreas	Immunocompetent subcutaneous cell line-derived human lung xenograft or syngeneic pancreas
Lopez et al., 2009 [30]	Female mice	Melanoma and pancreas	Immunocompromised subcutaneous cell line-derived xenograft (human tumour and stromal cells)
Freedman et al., 2018 [25]	Animal models were not used	Malignant ascites and prostate	N/A
De Sostoa et al., 2019 [31]	Female mice	Lung and pancreas	Immunocompromised subcutaneous cell line-derived xenograft (human lung or pancreas)
Harryvan et al., 2022 [32]	Male and female mice	PDAC	Immunocompetent subcutaneous syngeneic model
Kurisu et al., 2021 [33]	Female mice	Melanoma	Immunocompetent subcutaneous syngeneic model
Mistarz et al., 2023 [34]	Female mice	Ovarian	Immunocompetent orthotopic syngeneic model

In terms of TME representation, murine and human tumours grow in the orders of weeks and years respectively such that levels of immune suppression and TME heterogeneity in mice fail to recapitulate those in humans. Future studies would therefore benefit from the use of patient-derived xenografts (PDX) directly from human biopsies, allowing for better maintenance of the heterogeneity of tumour cells and CAFs, forming a more representative murine model. These should be administered orthotopically to more accurately recapitulate the TME for the specific tumour type, unlike the frequently used subcutaneous engraftments. A recent study using orthotopic syngeneic ovarian cancer murine models indicated that the TME replicated that of human tumours [49], indicating a lack of need for PDX. However, this study did not distinguish murine from human CAFs, which differ phenotypically [50], thus rendering syngeneic models unrepresentative. Overall, future studies should utilise orthotopic humanised PDX models, providing a better representation of the efficacy of CAF-targeting OVs.

The sex of mice used is often overlooked. Most of the CAF-targeting OV studies made use of solely female mice (Table 3). Although there is little literature surrounding sex-differences in murine CAFs, a recent study has shown murine male cardiac fibroblasts are more susceptible to inflammatory stimuli and hence activate in pro-inflammatory environments, upregulating both protumoural TGF-β and antitumoural IL-1β [51]. This sex-difference may play a role on immune-suppression as fibroblasts transition to CAFs. Furthermore, in human prostate cancer, oestrogen has been implicated as protumoural, acting via the G-protein estrogen receptor (GPER) on CAFs to metabolically switch them to a glycolytic state, providing paracrine lactate to tumour cells that leads to breast cancer progression [52]. Oestrogen has also been shown to promote mobilisation of bone marrow-derived precursors of CAFs to the TME in human breast cancer, increasing CAF-density [53]. Therefore, an antitumoural effect is more likely to be seen in pre-menopausal women with a larger pool of protumoural CAFs present, potentially biasing results on CAF-targeting OVs. For these reasons, studies should utilise male mice and stratify antitumoural responses according to sex, enabling sex-dependent differences to be elucidated.

Although it is normal to initially trial therapies in animal studies, there is a big gap between the best data in existing animal models and the patient population. This is because even humanised PDX murine models tend to lack the tumour heterogeneity and level of immune suppression seen in patients. Stromal architecture is also altered, with human CAFs being rapidly replaced by murine CAFs [54]. Wollenberg et al. [55] highlighted the differing effects of OV therapy on mice and human cells, with reovirus unable to induce immunogenic cell death in human tumour cells due to lack of RIPK1 expression yet it could in murine fibroblasts. CAF-targeting OVs are thus likely to have different efficacies when testing in murine models compared to humans in trials. To bridge the gap between animal studies and human trials, primary human tumour biopsies [25] and primary human tumour organs [56] maintained *ex vivo* should be utilised. These retain the full stromal, immunosuppressive and heterogenous landscape seen in human tumours, incorporating the diversity of TMEs across different patients and tumour types to better mimic clinical scenarios. As such, these are more representative to treat than murine models and thus will allow the testing of CAF-targeting OVs to a greater level, providing optimal preclinical data. One should not overlook the utility of alternative models such as organoids and 3D culture models as methods of more accurately simulating human tumour environments. Such technology generates three-dimensional tissue cultures which are crafted to replicate the dimensions, genetic profile and heterogeneity of source tumours in patients [57], allowing for a more comprehensive assessment of the cellular state and interactions between cell types. These could be used alongside *ex vivo* human tumour biopsies and organs in order to provide an array of data that could give greater insight into CAF-targeting OVs and their efficacy.

#### 1.2.4 Tumour types

A limited number of tumour types have been used to investigate CAF-targeting OVs (Table 3), mainly pancreatic, prostate and breast cancers. These tend to be cancers with the greatest abundance of CAFs [58]. As such, the effects of depleting CAFs in preclinical models are more likely to appear “antitumoural” if there is a larger reservoir to be depleted, putting to question CAF-targeting in cancer subtypes with fewer CAFs. Freedman et al. [25] highlighted this, whereby the malignant ascites biopsy with the lowest CAF:cancer cell ratio showed antitumoural effects due to direct virolysis of tumour cells rather than depletion of CAFs. This was evidenced through excessive OV-specific gene changes in both control and FAP-BiTE expressing EnAd OV treatment [25]. This may indicate these OVs for the treatment of CAF-abundant tumours only. Overall, this advises future studies to investigate the role of these OVs in a wider range of tumours to assess the applicability of this treatment to more tumour types, highlighting which patients would benefit more from CAF-targeting OVs.

### 1.3 Future prospects

Current preclinical studies, whilst promising and demonstrating a reduction in tumour growth, do not show complete responses. Several considerations could be made to enhance outcome in future studies. Firstly, understanding of CAF-subtype biomarkers can lead to more specific CAF-targeting, depleting only pro-tumourigenic subtypes to better elicit antitumour responses. Several challenges need to be overcome before this can be achieved. Literature regarding CAF biology is currently limited, and as such makes it difficult to identify and characterise pro- and antitumoural CAFs. For example, myCAFs have been shown in differing studies to exhibit pro- and antitumoural properties [38,39], the reasoning behind which being unclear. CAFs interact in a multifaceted way with the tumour milieu, providing a challenge for predicting the properties of CAFs both within and between patients. To unleash the full potential of CAF-targeting OVs, greater exploration of the distinct phenotypic and functional properties of subpopulations of CAFs is needed, as well as how they adapt to differing TMEs in various cancer types and stages. As CAFs are likely to exhibit heterogeneity across patients, this reduces the likelihood of utilising a single surface marker associated with a protumoural CAF phenotype for targeting by oncolytic virotherapy. This approach may mediate off-target effects on antitumoural CAFs as well as prove futile in patients with a similar protumoural CAF phenotype but lack the surface marker, thus advocating for a personalised approach. Establishing understanding on the nature of CAFs and their spatial and temporal changes in TMEs across patients through preclinical study is warranted to further therapy and bolster antitumoural responses.

Innovative technological advancements could also be utilised to improve the precision of OV targeting. Whilst off-target effects have not been reported for CAF-targeting OVs, they are widely recognised for other OVs [59,60], implying that they will likely pose a challenge in CAF-targeting OV therapy. Nanoparticle delivery systems have been trialled to overcome this, as shown by Li et al., 2024 [61] whom utilised OV-like nanoparticles to release nucleic acid drugs into tumour cells. This was done by using OVs as nanocarriers which release nanoparticles into the cytoplasm of cells only in response to high concentrations of glutathione, which were found specifically in tumour cells and not normal cells. These nanoparticles were adapted to deliver RNA for immunostimulatory agents in a murine melanoma model, with tumour regression identified. This could be adapted for CAF-targeting OVs through analysis of the cytoplasm content of different CAF subtypes, enabling specific protumoural CAF targeting and minimising off-target effects such as those against antitumoural CAFs. Furthermore, in recent years, CRISPR-based gene editing has emerged as a more efficient way of manipulating viral genomes. This has overcome the laborious, multi-step and inefficient methods currently used, largely utilising the homologous recombination system based on bacteria. Studies in HSV-1 OVs have shown that CRISPR-based gene editing had an efficiency of 8.41 % in inserting an EGFP reporter gene compared to 0.0000015 % using non-CRISPR homologous recombination [62]. This makes CRISPR-based gene editing an attractive future option for modification of CAF-targeting OVs to enhance their tropism for the specific CAFs they target, minimising off-target effects and furthering efficacy.

**Fig. 3:**
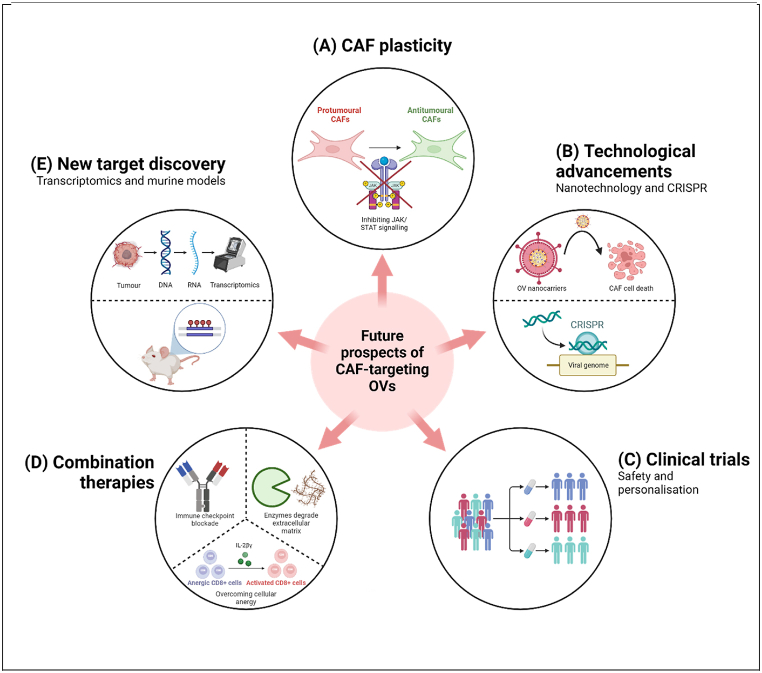
– Five prospective strategies for improving future CAF-targeting OV therapies. These are discussed in further detail below. (A) CAF plasticity can be exploited through the use of different signalling pathways to convert protumoural CAFs into an antitumoural phenotype, an example of which could be through inhibiting JAK/STAT signalling in protumoural CAFs. (B) Technological advancements in the form of nanotechnology and CRISPR can be utilised to increase the precision of OV targeting of CAFs, reducing off-target effects and furthering efficacy. (C) Future clinical trials are required in order to assess the safety of CAF-targeting OVs, as well as analysing patient tumoural landscapes in order to enable personalisation of therapy, both of which aim to improve treatment outcomes. (D) Combination therapies will likely play a key role in this field to synergise with the OV therapy to overcome the immunosuppressive TME. This will involve therapies such as immune checkpoint blockade, enzymes to degrade immunosuppressive ECM proteins, and cytokines to reactivate immune cells from an anergic state. (E) The identification of future targetable biomarkers and signalling pathways is essential for increasing our understanding of how to better target CAFs, particularly protumoural ones. These targets could be identified through multiple approaches, including the study of genetically modified murine models and transcriptomic analysis of CAFs from patient tumour biopsies. Created in BioRender. Al-obaidi, I. (2024) BioRender.com/u27b713.

CAF plasticity also has potential to be exploited therapeutically, promoting the conversion of protumoural CAFs into antitumoural ones (Fig. 2, Fig. 3). Studies in PDAC murine models have shown that janus kinase (JAK) inhibitors shift the protumoural iCAF phenotype to myCAFs, resulting in reduced tumour growth [63]. Molecular mediators that induce this conversion can be simultaneously administered or expressed by CAF-targeting OVs, such as expressing anti-IL-1α blocking antibodies that inhibit the janus kinase/signal transducer and activator of transcription (JAK/STAT) signalling pathway [63]. This both depletes immunosuppressive CAFs and converts remaining CAFs to an antitumoural phenotype, enhancing efficacy to increase the likelihood of complete response.

Murine models may be used to uncover novel targetable CAF-related signalling pathways. Identification of CAF-subtype specific promoters could lead to CAF-specific inducible Cre-lox murine models, with tumours induced via another recombination system such as FLP-FRT. These models can be used for temporal-specific knockout of the expression of CAF genes thought to be involved in protumoural CAF functions, towards ultimately understanding potential genetic or protein targets. Nonetheless, off-target knockouts will need to be considered when accounting for the shift in phenotype, given observations such as recombination of smooth muscle cells in fibroblast-specific Cre driver lines [64]. Cre-lox can further be used in reporter systems for lineage tracing of CAFs. Identified CAF-precursors could then be targeted by OVs, which may be important in preventing metastasis as CAFs can aid in forming the pre-metastatic niche by providing a protumoural environment along with circulating tumour cells. This could serve to control wide-spread dissemination of tumours.

As our understanding of CAF subpopulation biomarkers and signalling pathways improves with preclinical modelling, this may inform clinical trial design. Patient tumour biopsies could be utilised to stratify patients to responder and non-responder groups for CAF-targeting OVs. This would be through identification of anticipated markers of response, including pro- and antitumoural CAF biomarkers, their expression levels and CAF density. Caution is however warranted, as it remains unclear how different CAF types impact on tumour progression. Nevertheless, emerging technologies such as spatially resolved transcriptomics and scRNA-seq, as well as other -omic approaches, could then be employed to enable precise characterisation of patient CAF molecular landscapes, as CAFs are likely to be patient-specific due to tumour and CAF heterogeneity. This would permit personalisation of CAF-targeting OVs, exploiting patient-specific pathways, such as through using these OVs as delivery vectors for short hairpin RNAs [65] that restrict CAF-specific protein expression. Furthermore, sequencing patient tumours would enable the identification of the dominant pro-tumoural CAF subtypes for targeting, enabling a more personalised approach for CAF-targeting OVs to enhance efficacy.

Whilst efficacy and tumour regression are vital outcome measures, in these early stages, it is paramount that patients are monitored for adverse effects. OVs generally have a tolerable safety profile, with currently approved OVs such as Talimogene laherparepvec (T-VEC) exhibiting largely grade one and two adverse events, predominantly transient flu-like symptoms [66]. Despite this, there are potential biosafety issues that currently remain unclear due to the novelty of OVs as a therapy. These include potential long-term latent infection, shedding and transmission to contacts of patients and their indications in pregnant patients [67]. Methods to address these will need to be developed to optimise the safety of OVs as a therapeutic treatment. Further to this, the side effects of targeting CAFs are currently significantly underreported preclinically and yet to be observed in the clinical setting, and as such may yield unknown effects. Deep genomic sequencing of patient tumours and associated CAF populations would permit dissection of signatures associated with any adverse effects, allowing for informed clinical decision making through risk-benefit assessment.

Importantly artificial intelligence (AI) and machine learning (ML) may facilitate future development and implementation of CAF-targeting OVs, towards the goal of personalised immunotherapy. Specificity of CAF-targeting OVs and selection of appropriate delivery platforms may be enhanced through utilisation of AI to analyse multi-omic data sets to identify biomarkers associated with protumoural and antitumoural CAFs, or perform cellular image analysis of tumour biopsies to characterise features of the tumour microenvironment prior to and following OV therapy [68]. Furthermore, signatures of treatment-responsive patients may be elucidated using ML models which synthesise data including patient history, demographics, genetic, imaging and response from clinical trials. Ultimately, this may lead to the development of supervised ML algorithms, which can predict optimal immunotherapy and dosage based on stratification from existing patient data, enabling clinicians to make better-informed decisions according to individualised-risk benefit. This may also enable avoidance of treatment-related toxicities, while AI monitoring of patient results at checkups may facilitate dynamic treatment planning and identification of early response signatures. Critically, data sets upon which AI/ML models are trained must represent the demographic diversity of populations that are to benefit from CAF-targeting oncolytic virotherapy, to improve predictive validity and enable equitable treatment benefit [69].

Moving forward, it will be important to characterise the long-term impact of these therapies. Current studies have highlighted potential mechanisms of immunoediting to overcome these CAF-targeted therapies. This includes downregulation of the pathway being targeted by the OVs, as seen with SPARC promoter downregulation [30], alongside concomitant upregulation of immune checkpoint ligands [25]. This may indicate the use of combinatorial approaches to target multiple pathways. For example, immune checkpoint blockade can be utilised, either through administration of ICB drugs or engineering CAF-targeting OVs to express ICB antibodies, to overcome the immune suppression provided by checkpoint markers. This may require destruction of protumoural CAFs, as these have been found to correlate with poor responses to ICB [36], namely LRRC15^+^ CAFs, through a potential immunomodulatory role. It is key to note that the effect of combining ICBs with OVs is bidirectional, with a study highlighting that OVs promote ICB efficacy through increasing intratumoural T-cell infiltration into immunologically “cold” tumours. Another approach to circumvent immune evasion mechanisms may be to simultaneously arm CAF-targeting OVs to target other aspects of CAF biology, such as producing enzymes which degrade protumoural ECM protein components produced by CAFs. Studies [70,71] have highlighted the potential of this approach in pre-clinical mouse models, showcasing enhanced OV dissemination and modification of the immune landscape to be permissive to antitumoural cells. This was found to improve the outcomes of current cancer therapeutics, including chemo- and immunotherapies, and hence may prove to have similar synergistic effects with CAF-targeting OVs. Overall, future clinical trials would benefit from exploring a variety of potential combinatorial approaches to enhance long-term immune responses, and hence treatment outcomes and prognosis. Furthermore, doubts to long-term antitumoural efficacy have been implicated: Mistarz et al. [34] isolated CD8^+^ T-cells from an immunosuppressive CAF environment after depletion of CAFs and tumour regression and found that these had reduced cytotoxic activity compared to wild-type CD8^+^ T-cells from depleted immunostimulatory CAF TMEs, emphasising potential long-term dysfunction. Overcoming this anergy will be key for enhancing efficacy of these CAF-targeting OVs. This could include co-administration of recombinant cytokines such as IL-2βγ to activate IL-2βγRs expressed on exhausted CD8^+^ T-cells [72]. Additional multi-omic profiling of these CD8^+^ T-cells may identify avenues for reprogramming specific molecular mechanisms to support activation, including epigenetic regulators as combination therapy [73]. Furthermore, other components of the TME may also impact on the efficacy of CAF-targeting OVs. For example, studies in colon cancer cell lines [74] have shown that IL-6 and IL-8 released by CAFs upregulates myeloid-derived suppressor cells, and hence is associated with immunosuppression. Similar studies in hepatocellular carcinoma have shown CAF upregulation of regulatory dendritic cells which produce inhibitory cytokines and enzymes such as indoleamine 2,3-dioxygenase [75]. These immunoregulatory cells within the TME may limit antitumoural responses induced by CAF-targeting OVs, and as such warrant investigation into potential simultaneous targeting to mediate greater therapeutic efficacy. Altogether, all these considerations will allow for greater optimisation of CAF-targeting OV therapies in enhancing antitumoural immune responses.

## 2 Conclusion

Despite the first CAF-targeting OV going ahead for clinical trial, several features of experimental design of the studies in this field make it difficult to assess the likelihood of success of this therapy in transitioning to the clinic. Efforts should be made in improving pre-clinical analysis of CAF function and modelling systems in view of the limitations of pre-clinical studies, allowing for greater applicability to humans and improved guidance for how therapy should be approached. We suspect that, even after optimising pre-clinical data, clinical trials will indicate differing antitumoural immune responses between patients due to TME heterogeneity, as has been observed with previous immunotherapies. This likely differential efficacy of CAF-targeting OVs on specific tumour types will serve to fuel the imminent precision medicine revolution. Undoubtedly, CAF-targeting OVs represent a promising new cancer therapeutic, with their full potential yet to be uncovered.

## CRediT authorship contribution statement

**Ibrahem Al-Obaidi:** Writing – review & editing, Writing – original draft, Project administration. **Ciaran Sandhu:** Writing – review & editing. **Bilal Qureshi:** Writing – review & editing. **Leonard W. Seymour:** Writing – review & editing, Supervision, Project administration, Conceptualization.

## Ethics statement

The authors declare that this article abides by Heliyon's Ethics and Editorial Policies.

## Data availability statement

No data was used for the research described in this article.

## Funding

The authors received no financial support for the research, authorship and publication of this article.

## Declaration of competing interest

None.
